# Supplementary material for: Coherent Motion of Monolayer Sheets under Confinement and Its Pathological Implications
Source: PLoS Comput Biol. 2015 Dec 21;11(12):e1004670. doi: 10.1371/journal.pcbi.1004670 (PMC4686989; doi:10.1371/journal.pcbi.1004670)
Supplement: S1 Text — (PDF) [file pcbi.1004670.s001.pdf]

Coherent motion of monolayer sheets under confinement and its pathological implications

S S Soumya, Animesh Gupta, Andrea Cugno, Luca Deseri, Kaushik Dayal, Dibyendu Das, Shamik Sen, and Mandar M. Inamdar

SUPPORTING INFORMATION

Text S1. RELATION BETWEEN SPRING CONSTANT (k) AND YOUNG'S MODULUS (E) FOR TRIANGULAR NETWORK OF SPRINGS

Let us consider a triangular network of springs with A, B, C as the initial position of cells and a_0 as the length of springs at equilibrium as shown in Fig. S1. Now, each cell is given a uniform stretch δ so that their position has changed to A', B' and C' respectively. For a network with six fold symmetry, the potential energy change associated with this stretching can be written as $\Delta F = 3 \times \frac{1}{2}k\delta^2$[1], where k is the spring constant. Assuming the system as elastic, isotropic and homogenous, the potential energy stored in the system as the result of deformation is equal to the strain energy of the system which can be written as $U = \frac{1}{2}\sigma_{ij}\epsilon_{ij}V$, where σ_{ij} is the stress tensor, ϵ_{ij} is the strain tensor and V is the volume of the element. Assuming the system as elastic and isotropic, the stress strain relationship can be written as $\epsilon_{ij} = \frac{(1+\nu)}{E}\sigma_{ij} - \frac{\nu}{E}\sigma_{kk}\delta_{ij}$, where E denotes the Young's modulus, ν is the poison's ratio and δ_{ij} is the unit tensor. For an isotropic stretch for a 2-D system, the normal strain components will be equal and shear will be zero. So $\epsilon_{xx} = \epsilon_{yy} = \epsilon = \frac{\delta}{a_0}$ which will give the stress components as $\sigma_{xx} = \sigma_{yy} = \sigma = \frac{E}{(1-\nu)}\frac{\delta}{a_0}$. Now the total strain energy of the system will be $U = \frac{E}{(1-\nu)}(\frac{\delta}{a_0})^2 V$. Writing volume V in terms of the height of the element, h and equilibrium length of spring a_0 as $V = \sqrt{(}3)/4 \times a_0^2 h$, and equating strain energy with the change in potential energy, the spring constant will be $k = \frac{Eh}{2\sqrt{3}(1-\nu)}$

[1] Boal D. Mechanics of the Cell. Cambridge University Press; 2012.
