# Supplementary material for: Coherent Motion of Monolayer Sheets under Confinement and Its Pathological Implications
Source: PLoS Comput Biol. 2015 Dec 21;11(12):e1004670. doi: 10.1371/journal.pcbi.1004670 (PMC4686989; doi:10.1371/journal.pcbi.1004670)
Supplement: S2 Text — (PDF) [file pcbi.1004670.s002.pdf]

Coherent motion of monolayer sheets under confinement and its pathological implications

S S Soumya, Animesh Gupta, Andrea Cugno, Luca Deseri, Kaushik Dayal, Dibyendu Das, Shamik Sen, and Mandar M. Inamdar

SUPPORTING INFORMATION

Text S2. WHY IS COHERENT ROTATIONAL MOTION SEEN FOR A SELF-PROPELLED ELASTIC SOLID WHEN THE POLARIZATION VECTOR FOR A CELL HAS A TENDENCY TO ALIGN WITH THE VELOCITY OF THE CELL?

The equation of evolution for cell position and polarization, respectively, for the current system are (also see main text):

$$\frac{d\boldsymbol{r}_i}{dt} = v_0\hat{\boldsymbol{p}}_i + \mu\boldsymbol{F}_i,$$
$$\frac{d\hat{\boldsymbol{p}}_i}{dt} = \xi(\hat{\boldsymbol{p}}_i \times \hat{\boldsymbol{v}}_i.\boldsymbol{e}_z)\hat{\boldsymbol{p}}_\perp \tag{S1}$$

As per this evolution rule, the polarization of the cell has a tendency to align with its velocity. Additionally, the polarization direction also feeds into the velocity of the cell and tends to modify its speed and direction. Now, to understand, at least semi-analytically, the origin of the rotational motion under confinement for an elastic solid formed of self-propelling particles as given above, we use the following procedure motivated from the arguments in Refs. [1, 2]. We extend this reasoning to argue that even if the cells can exchange their neighbors, coherent rotational motion of the cell sheet is the most likely outcome.

The elastic system of springs under circular confinement is free to undergo rigid body rotation about its center. Its translational degrees of freedom are, however, curtailed due to the confinement. Using ideas from structural mechanics, let us denote the stiffness matrix of the system in its rest (or stress-free) conformation as $[K]$ [3]. The matrix $[K]$, is symmetric, positive semi-definite, and has dimensions of $2N_{\text{cells}} \times 2N_{\text{cells}}$. If the displacement of the cells (nodes) from their rest position, in terms of column matrix of dimension $2N_{\text{cells}} \times 1$, is $\{u\}$, then the equation of motion for the system in the complete matrix form can be written as:

$$\frac{d\{u\}}{dt} = v_0\{p\} - \mu[K]\{u\}. \tag{S2}$$

Additionally, in this case, the polarization of each cell prefers to align with its velocity (see Eq. S1). Since the stiffness matrix $[K]$ is symmetric and positive semi-definite, it has $2N_{\text{cells}}$ orthogonal eigenvectors $\{\phi_i\}$ and corresponding non-negative eigenvalues λ_i. The only zero eigenvalue is the one corresponding to the rigid body rotation mode $\{\phi_0\}$; all other eigenvalues are positive. The displacement $\{u\}$ and velocity $\{\dot{u}\}$ can then re-written

in the form of eigen-modes as

$$\{u\} = \sum_{i=0}^{2N_{\text{cells}}-1} \alpha_i \{\phi_i\}, \text{ and} \tag{S3}$$

$$\{\dot{u}\} = \sum_{i=0}^{2N_{\text{cells}}-1} \dot{\alpha}_i \{\phi_i\}, \tag{S4}$$

where $\{p\}$ is the polarization of all the cells, combined in the form of a column vector of size $2N_{\text{cells}} \times 1$. Expressing Eq. S2 using the eigenmodes, we get the following set of equations in terms of eigenmode amplitudes

$$\frac{d\alpha_j}{dt} = v_0 \langle \phi_j \rangle \{p\} - \mu \lambda_j \alpha_j, \tag{S5}$$

where $\langle \phi_i \rangle$ is the eigenvector written in row format. This equation can be re-written as:

$$\frac{d\alpha_j}{dt} = v_0 \beta_j - \mu \lambda_j \alpha_j, \tag{S6}$$

where $\beta_j = \langle \phi_j \rangle \{p\}$. It seems safe to presume that for a certain time interval $\tau \sim 1/\xi$, where ξ is the response rate for polarization (see Eq. S1), the polarization remains almost constant. In this case Eq. S5 can be solved to provide us the following solution for the amplitude α_j of any mode j.

$$\alpha_j = \frac{v_0 \beta_j}{\lambda_j \mu} [1 - \exp(-\mu \lambda_j t)], \tag{S7}$$

and the corresponding *velocity* of the mode is given by

$$\dot{\alpha}_j = v_0 \beta_j \exp(-\mu \lambda_j t), \tag{S8}$$

where we have assumed zero initial conditions for α_j. It is very clear from Eq. S8 that modes with larger λ, i.e., greater stiffness or small wavelength, would decay faster as compared with the modes with smaller λ. The smallest λ possible for the current system is $\lambda_0 = 0$, corresponding to the mode that involves rigid body rotation of the tissue. This implies that the amplitude α_0 corresponding to pure rotation, and more importantly the angular speed $\dot{\alpha}_0$ ($v_0 \beta_0$) increases with time. The motility parameter μ sets the rate at which the energy is dissipated from mode $j \neq 0$, whereas the polarization orientation constant ξ sets the rate at which the energy is pumped into each mode (in the form of β_j).

Let us first look at the case with *medium* value of $\xi \approx 1$, i.e., $\xi \approx \mu \lambda_1$. The polarization column vector $\{p\}$ for the system can also be written in terms of eigen-modes as follows:

$$\{p\} = \sum_{i=0}^{2N_{\text{cells}}-1} \beta_i \{\phi_i\}, \tag{S9}$$

As can be seen from Eq. S8, for the case of $\xi \approx \mu\lambda_1$, the velocity component corresponding to ϕ_0 increases, whereas the components corresponding to other modes essentially decay to zero—in the very least they do not grow as fast as α_0. It may be noted that, since, the polarization vector for each cell has unit magnitude, the consolidated column vector will additionally satisfy

$$\langle p\rangle\{p\} = \sum_{i=0}^{2N_{\text{cells}}-1} \beta_i^2 = N_{\text{cells}}. \tag{S10}$$

Hence, the fact that $\dot{\alpha}_0$ is the dominant mode will ensure that β_0 will increase to some bounded steady state value that would depend on the polarization orientation parameter ξ, since, as per our polarization rule the polarization of a cell tends to align with its velocity. It may, however, be noted that since the polarization of each cell is a unit vector, in addition to a dominant β_0, some other β_i components would also remain non-zero. This means that, as per Eq. S6, some energy will keep getting pumped in a few other modes i. Nonetheless, $\dot{\alpha}_0$ will be the only component that would increase steadily as per Eq. S8—other components $\dot{\alpha}_i$ would decay.

We now examine two extreme limits of polarization orientation constant ξ. When ξ is very small($\xi << 1$), the response of $\hat{\mathbf{p}}$ to velocity $\mathbf{v}$ is slow. As a result, the polarizations of the cells would lag behind in their bid for orienting with the velocity (see Fig. 2(a), (b), (d) of the main paper), resulting in a smaller steady state value for β_0. However, as described above, even a small component β_0 of the polarization field would be sufficient to sustain steady $\dot{\alpha}_0$, and hence rotation—the other modes $\dot{\alpha}_j$ would not be sustained despite having non-zero β_j values in some of the modes. The angular velocity of the tissue ($\omega \approx v_0\beta_0$) would be, of course, small as is seen in Fig. 2(c). In the other extreme limit when $\xi \gg 1$, the rate at which energy is pumped in the modes is faster than the rate at which it is typically dissipated for the jth mode ($\sim \mu\lambda_j$). In this case, we can see (Video S3), a perfect rotation of the tissue about the center is not obtained—the centre of rotation keeps changing constantly, confirming that $\{\phi_0\}$ is not the only mode that is invoked. Indeed, as can be seen from Video S3, a few radial modes are also excited, and are reminiscent of such movements observed in Ref. [4]. Our calculation predicts that, if the cells are highly cohesive, in which case their polarization can evolve faster [5] ($\xi \gg 1$), we are expected to see these non-rotational, radial, modes. Similarly, since the stiffness of the long wavelength modes (λ_j) is inversely proportional to the system size (e.g., in $1-D$, $\lambda_j \sim j^2/L$), we can see such modes for larger

system even if $\xi \approx 1$. In fact, such movements are also reported in the experiments of Deforet et al [4] and the simulations of Ref. [6] for larger system sizes—the authors attribute these modes to the lack of the strength of persistent force (v_0/μ in our case). Our calculation, however, gives a clearer understanding for the origin of these movements.

The previous argument hinged on the tissue having a well defined stiffness matrix $[K]$, which in turn depends on having a system of cells with fixed connectivity. However, in our model we allow for the cells to change their neighbors and release internal stress. If that happens, the stiffness matrix of the tissue gets modified to $[K']$, depending on the rate at which the cells change their neighbors. As a result, the eigenvectors of the system now get modified to $\{\phi_i'\}$. The only eigenvector that is, however, most certainly common to the two systems is the one corresponding to the rigid body rotation $\{\phi_0\} = \{\phi_0'\}$. Consequently, though there will be perturbations to β_i in the form of new β_i', the steady pumping of energy to the rotational mode will continue. The system is, hence, expected to achieve rotational coherence even if the cells (nodes) are allowed to change neighbors. Though this argument is not rigorous, it is consistent with the results of our simulations, and indeed seems plausible.

There are a couple of things that we did not account in the above derivation: (i) pre-stress in the system due to crowding, and (ii) finite rotation effects. The pre-stress effect is too complex to be accounted for in this simple derivation—we leave it for future work. The effect of finite rotation seems to be a secondary effect. We see (Video S1) that when the cell sheet, by and large, behave elastically the coherent rotation is initiated before any finite rotation actually happens in the system. As a result, we think that the finite rotation effect is secondary, and if required can be incorporated by moving to a co-rotational frame of reference (as is done in section deriving the analytical solution for elastic solids in main text)—it should not affect essential mechanics of the problem.

---

[1] Ferrante E, Turgut AE, Dorigo M, Huepe C. Collective motion dynamics of active solids and active crystals. New J Physics. 2013; 15(9):095011.

[2] Henkes S, Fily Y, Marchetti MC. Active jamming: self-propelled soft particles at high density. Phys Rev E. 2011; 84(4):040301.

[3] Leet K, Uang CM, Gilbert AM. Fundamentals of structural analysis. McGraw-Hill; 2002.

[4] Deforet M, Hakim V, Yevick H, Duclos G, Silberzan P. Emergence of collective modes and tri-dimensional structures from epithelial confinement. Nat Commun. 2014; 5:3747.

[5] Vitorino P, Meyer T. Modular control of endothelial sheet migration. Genes Dev. 2008; 22(23):3268–3281.

[6] Li B, Sun SX. Coherent motions in confluent cell monolayer sheets. Biophys J. 2014; 107(7):1532–1541.
