# Supplementary material for: Coherent Motion of Monolayer Sheets under Confinement and Its Pathological Implications
Source: PLoS Comput Biol. 2015 Dec 21;11(12):e1004670. doi: 10.1371/journal.pcbi.1004670 (PMC4686989; doi:10.1371/journal.pcbi.1004670)
Supplement: S3 Text — (PDF) [file pcbi.1004670.s003.pdf]

Coherent motion of monolayer sheets under confinement and its pathological implications

S S Soumya, Animesh Gupta, Andrea Cugno, Luca Deseri, Kaushik Dayal, Dibyendu Das, Shamik Sen, and Mandar M. Inamdar

SUPPORTING INFORMATION

Text S3. EXACT STEADY STATE SOLUTION WHEN THE TISSUE IS A VISCOUS FLUID

Since, in our model, the cells are allowed to change neighbours and relax the internal stress, then depending on the internal strain/stress, the tissue can indeed behave more like a fluid than like an elastic solid as described in the main paper. We hence, present a simple semi-analytical case of a Newtonian fluid to demonstrate coherent rotation for a fluidised tissue, and resort to the simulation results to make any contact with experiments. A more realistic, description of the tissue as a complex fluid is beyond the scope of this paper, due to the difficulty in both, using an appropriate rheological model, as well as in obtaining analytical solutions.

In this case, we seek to obtain a radially symmetric solution such that both the polarisation $\hat{\mathbf{p}}$ and velocities are aligned along the tangential direction (i.e., $v_r = 0$). To do so, we write a very simple form for the equation of equilibrium as is given below. The equation for polarisation evolution remains the same as before (Eq. S1), and if we can find a such a solution, then we have found one possible steady state solution.

The constitutive equation for the epithelial sheet that is modelled as a viscous fluid is written as

$$\sigma = \eta \left(\nabla \mathbf{v} + (\nabla \mathbf{v})^T - (\nabla \cdot \mathbf{v})\mathbb{1} \right) + \eta_v (\nabla \cdot \mathbf{v})\mathbb{1}, \tag{S11}$$

where η is the $2-D$ shear viscosity, and $\eta_v = 3\eta$ in 2−D [1]. The equation of equilibrium in radial direction will be trivially reduced to zero for the solution that we are looking for. In the θ direction, the equation for tangential velocity v_θ will become,

$$2\eta \left(\frac{1}{r}\frac{\partial}{\partial r}\left(r \frac{\partial v_\theta}{\partial r} \right) - \frac{v_\theta}{r^2} \right) + \frac{1}{\mu_s}(v_0 - v_\theta) = 0, \tag{S12}$$

with the boundary conditions

$$v_\theta(0) = 0, \text{ (symmetry)} \qquad \frac{\partial}{\partial r}\left(\frac{v_\theta}{r} \right)_R = 0 \text{ (shear traction at boundary)}. \tag{S13}$$

This equation seems to have a complicated closed-form solution in terms of Bessel and Hypergeometric functions. However, we can solve this problem numerically. To do that we

will first non-dimensionalize the equation: all velocities are expressed in terms of v_0 and all lengths in terms of R. The equation then simplifies to

$$\frac{1}{r}\frac{\partial}{\partial r}\left(r\frac{\partial v_\theta}{\partial r}\right) - \frac{v_\theta}{r^2} + \alpha(1 - v_\theta) = 0, \qquad \left(\alpha = \frac{R^2}{2\mu_s\eta}\right). \tag{S14}$$

The quantity $\sqrt{\mu_s\eta} = R_h$ is the hydrodynamic length [1], and the ratio α is the relative size of confinement disc (R) with respect to the hydrodynamic length R_h. The non-dimensional Eq. S14 equation can be easily solved numerically. For low values of α the solution seems to be very similar to a rigid body rotation. When α becomes larger, the velocity initially increases with r and then saturates to v_0. One such plot for v_θ with respect to r is shown in Fig. S2.

[1] Kumar KV, Bois JS, Jülicher F, Grill SW. Pulsatory patterns in active fluids. Phys Rev Lett. 2014;112(20):208101.
