# Supplementary material for: Coherent Motion of Monolayer Sheets under Confinement and Its Pathological Implications
Source: PLoS Comput Biol. 2015 Dec 21;11(12):e1004670. doi: 10.1371/journal.pcbi.1004670 (PMC4686989; doi:10.1371/journal.pcbi.1004670)
Supplement: S4 Text — (PDF) [file pcbi.1004670.s004.pdf]

Coherent motion of monolayer sheets under confinement and its pathological implications

S S Soumya, Animesh Gupta, Andrea Cugno, Luca Deseri, Kaushik Dayal, Dibyendu Das, Shamik Sen, and Mandar M. Inamdar

SUPPORTING INFORMATION

Text S4. PASSIVE CONFINEMENT IS ALSO CAPABLE OF INDUCING COHERENT ROTATION

In order to test whether cells can coherently rotate under passive confinements instead of geometrical confinements, simulations were performed with three initial positions of the 'active' cells: in the center of the circular pattern (Figs. S4 (a)–(c), Video S4–S5), in the periphery of the circular pattern (Figs. S4 (d)–(f), Video S6–S7) and in an annular geometry (Fig. S3, Video S8–S11). Consistent with our hypothesis, our simulations with varying mobility ratios of active and passive cells suggests that differences in physical properties of cells (i.e., motility and mobility) can indeed induce coherent angular motion (Fig. S3, Video S8 - S11). When the frictional properties of active and inactive cells were comparable (i.e., $\mu_{\text{active}}/\mu_{\text{inactive}}) = 1$, active cells were found to intercalate into the tissue and not exhibit any coherent motion. However, a 100-fold decrease in mobility of passive cell induced segregation of active cells due to inability of active cells to move the passive cells, and led to onset of coherent motion. This was even more clear for $\mu_{\text{active}}/\mu_{\text{inactive}} = 1000$ where the majority of the active cells remained stuck in their initial positions, i.e., either centrally (Fig. S4(c)), or peripherally (Fig. S4(f)) or along the annulus (Fig. S3(d)), and exhibited coherent angular motion. Together, these results indicate that passive confinement effected by differences in cell mechanical properties is sufficient to induce coherent rotation.
