# Supplementary material for: Coherent Motion of Monolayer Sheets under Confinement and Its Pathological Implications
Source: PLoS Comput Biol. 2015 Dec 21;11(12):e1004670. doi: 10.1371/journal.pcbi.1004670 (PMC4686989; doi:10.1371/journal.pcbi.1004670)
Supplement: S5 Text — (PDF) [file pcbi.1004670.s005.pdf]

Coherent motion of monolayer sheets under confinement and its pathological implications

S S Soumya, Animesh Gupta, Andrea Cugno, Luca Deseri, Kaushik Dayal, Dibyendu Das, Shamik Sen, and Mandar M. Inamdar

SUPPORTING INFORMATION

Text S5. DETAILED ANALYSIS TO UNDERSTAND MECHANISMS THAT GOVERN FLUIDISATION OF THE TISSUE DURING COHERENT ANGULAR MOVEMENT

We had addressed various parameters and mechanisms that can lead to fluidisation of the tissue in Section "Cell crowding leads to fluidisation of tissue." Here, we present a detailed analysis of those mechanisms. Note that in the discussion below we interchangeably use distortion and shear strain; distortion implies change in shape, and shape deformation is the hallmark of shear strain [1].

Si. Confinement contributes to the distortion of the tissue

In the absence of any compression, the approximate size of a single undeformed cell in a tissue, assuming the cell shape to be approximately circular, will be $\pi(a_0/2)^2$. As a result, the critical number of cells that can be approximately packed in a circle of radius R is $N_c \approx R^2/(a_0/2)^2$. Using the canonical values for our model, $R = 5$ and $a_0 = 1$, this number $N_c \approx 100$. Hence, in a loose sense, a tissue under this confinement with more than 100 cells is expected to have deformation. If the tissue were elastic and homogeneous, one would expect the confinement leading simply to uniform and isotropic compression [1]. However, due to its discrete nature, the tissue is susceptible to having shear deformations in addition. This is because, the circular confinement does not permit all the cells to have a preferred co-ordination number $z = 6$ [2], thus leading to distortion of the tissue, since the triangles formed by cell-cell connections (springs) cannot be all equilateral. The presence of motility forces further distorts the tissue by contributing additional shear strains. In the steady state of coherent rotation, we hence, expect the distortion of cell-connection triangles to depend on cell density. We will see below that, these intuitive observations are indeed consistent with the findings of our simulations.

As described in the main paper, the connectivity of cells is obtained by Delaunay triangulation [3]. We can plot histograms of the qualities of cell-connection triangles spanning

the tissue in Fig. S7. The quality of a triangle is defined as [4]:

$$Q = \frac{4\sqrt{3}A}{h_1^2 + h_2^2 + h_3^2}, \tag{S15}$$

where, h_1, h_2 and h_3 are the side lengths of any given triangle, and A is its area. The quality factor $Q = 1$ for an equilateral triangle (no distortion), and reduces with increasing distortion. It can be clearly seen from Fig. S7 (a) and (b) that the number of relatively distorted triangles $Q < 0.9$ (chosen arbitrarily) is larger for the case $N = 170$ when compared with the less crowded $N = 140$. The steady state shear strain is, hence, observed to be comparatively larger for the more crowded tissue.

Delaunay triangulation updates the connectivity of cells if the updated triangulation has fewer "skinny," i.e, distorted triangles [5]. Hence, in a statistical sense, a more distorted tissue has greater susceptibility for changing its connectivity, thus resulting in its fluidization.

Since Delaunay triangulations are dual to Voronoi tessellations [5] (Fig. 1(b) of the main paper), cell distortions can be equivalently but better explained by looking at the statistics of Voronoi (cell) edge length of the tissue in Fig. S8 (a) and (b) for $N = 140$ and $N = 170$. Cells connected to each other share an edge. When the cells update their connectivity via Delaunay triangulation, this edge grows in the topologically orthogonal direction while transitioning through a point, or a four-way vertex (see Fig. S9). Hence, in a statistical sense, a tissue with a substantial number of small edges is more susceptible to T1 transitions (or neighbour change). Since, it can be clearly seen from Fig. S8 that, in its steady rotating state, the number of very small Voronoi edges $e < 0.2$ (chosen arbitrarily) for $N = 170$ case, is quite large when compared with the case $N = 140$, we have another cue as to why the tissue is more prone to fluidisation for $N = 170$, when compared with $N = 140$.

Sii. Increase in cell-cell connection (spring) stiffness reduces distortion and makes the tissue resistant to fluidisation

Upon increasing the stiffness of springs, though the initial shear pre-strain is not expected to change much due to the kinematic confinement provided by the circular boundary, the additional distortion caused by the motile forces would clearly be decreased. The decrease in overall distortion of the tissue for $N = 170$ can be clearly seen from Figs. S7(c) and S8(c). Due to the relative lower distortion, as compared to a softer tissue, the tissue is more resistant

to neighbour changes and hence fluidisation.

Siii. Neighbour changes via Delaunay triangulation happen in local patches in a time-sequential manner and releases local shear strain in the tissue

For any configuration of cell centers, Delaunay triangulation, in essence, provides the cells with connectivity that produces the least number of distorted cell triangles. As demonstrated in Fig. S9 (also see SI Video S22), for $N = 170$, the neighbour exchange happens at local "hot spots," in a frequent, and time-sequential manner. A local T1 transition event release the local shear strains in the tissue, as and when the strain builds up, and a neighbour change can reduce the same. As also described in the SI section below, T1 transition happens in our model only if re-triangulation results in a tissue with lesser distortion. If we prevent the T1 transition from happening, by "locking" the connectivity of cells, i.e., by not updating the connectivity of the cells, then distortion starts building up at local patches (SI Fig. S10(b) for $N = 170$) and steadily increases. Updating the tissue connectivity by Delaunay triangulation, which leads to fluidisation of the tissue, prevents build-up of shear strain and corresponding stress in the tissue. Thus updating cell-cell connectivity via Delaunay triangulation is a mechanically very relevant way to release shear strains in a tissue. Note that "locking" the connectivity does not lead to building up of stress for $N = 140$ (SI Fig. S10(a) and SI Video S21) because any modification in connectivity would only increase distortion in the tissue.

Siv. Skewness of cell-cell connection length distribution is a key quantity that decides the susceptibility of the tissue to fluidisation

The description given in the previous section clearly demonstrates the correlation between the shear distortion caused in the tissue by confinement with fluidisation upon crowding in the tissue. However, this does not explain why fluidisation is seen for $N = 170$ and not for $N = 140$, though confinement related distortion is present in both cases (see Fig. S7). Below we clearly describe that though the presence of shear strain is necessary for T1 transitions in the tissue, in itself it is not sufficient. For T1 transition to happen it is important that the local distortion of cells reduces upon T1 transition. Indeed, for $N = 140$, there are triangles

with distortion comparable for the case $N = 170$ (see Fig. S7). What then is the possible reason for the stability of $N = 140$, with respect to T1 transitions, despite having distortion in triangles (albeit fewer numbers) that is comparable to $N = 170$? The resolution to this question, again, lies with the degree of confinement. For $N = 140$, since the density of cells is relatively lower, in addition to short edges we expect to find a chunk of long connections with length $\approx a_0 = 1$ at steady rotation state. The presence of such relatively long edges amongst short edges would lead to triangles of the type shown in Fig. S11(b). Though these triangles indeed have shear distortion, connectivity update will lead to triangles of even higher distortion. When the number of cells is increased, the proportion of longer edges decreases, resulting in triangles shown in Fig. S11(b) that are not as robust to triangulation update.

To quantify, the proportion of long connections to short connections in the tissue we obtain the quantified the skewness in the distribution of edges for $N = 140, 150, 160, 170$ given by Pearson's second coefficient Sk_2 given by [6]:

$$Sk_2 = 3\frac{(\text{mean} - \text{median})}{\text{standard deviation}}. \tag{S16}$$

As shown in Fig. S11(c), Sk_2 increases with cell crowding, i.e., greater the skewness in the connection distribution, greater the amount of fluidisation of the tissue. This correlation is borne out consistently in other situations where shear can happen in the absence of crowding.

1. Consider the case, when $R = 10$ and $N = 560$, this corresponds to cell density equivalent to $R = 5$ and $N = 140$. So, contrary to our exception, we indeed observe fluidisation of the tissue during steady rotation. This is very likely because the maximum shear in the tissue is proportional to R (see Eq. 14 and Fig. 5(a) of the main paper), as a result of which, the distortion due to motile forces can push the tissue over triangulation stability. The same behaviour is observed for $R = 15$ and $N = 1080$ (Fig. 5(a)). The index Sk_2 is around 0.4 for both cases, which is greater than the value of $Sk_2 = 0.3$ for $R = 5$ and $N = 140$ (no fluidisation).

2. If we increase the radius of confinement to $R = 5.8$ such that the cell density for $N = 170$ is similar to the case $R = 5$ and $N = 140$, we indeed observe that there is no fluidisation in the tissue. The value of Sk_2 reduces from 0.54 ($N = 170, R = 5$) to 0.16.

3. If the stiffness of the cell connections is increased to $k_c = k_t = 100$, the fluidisation of the tissue is prevented and the value of Sk_2 reduces drastically from 0.54 to 0.04. On the other hand, if k_t is reduced to 1, then even for $N = 140$ and $R = 5$ case, tissue undergoes fluidisation, and the value of Sk_2 increases from 0.3 for no fluidisation to 0.4.

To summarise the findings, we find that the skewness ratio of connection lengths is intricately linked with the susceptibility of tissue to fluidisation.

---

[1] Sadd MH. Elasticity: theory, applications, and numerics. Academic Press; 2009.

[2] Williams I, Oğuz EC, Jack RL, Bartlett P, Löwen H, Royall CP. The effect of boundary adaptivity on hexagonal ordering and bistability in circularly confined quasi hard discs. J Chem Phys. 2014; 140(10):104907.

[3] Mirams GR, Arthurs CJ, Bernabeu MO, Bordas R, Cooper J, Corrias A, et al. Chaste: an open source C++ library for computational physiology and biology. PLoS Comput Biol. 2013; 9(3):e1002970.

[4] Matlab 2012b Manual. The MathWorks, Inc, Natick, Massachusetts, United States. 2012;.

[5] De Berg M, Van Kreveld M, Overmars M, Schwarzkopf OC. Computational geometry. Springer; 2000.

[6] Doane DP, Seward LE. Measuring skewness: a forgotten statistic. J Stat Educ. 2011; 19(2):1–18.
