# Supplementary material for: Coherent Motion of Monolayer Sheets under Confinement and Its Pathological Implications
Source: PLoS Comput Biol. 2015 Dec 21;11(12):e1004670. doi: 10.1371/journal.pcbi.1004670 (PMC4686989; doi:10.1371/journal.pcbi.1004670)
Supplement: S6 Text — (PDF) [file pcbi.1004670.s006.pdf]

Coherent motion of monolayer sheets under confinement and its pathological implications

S S Soumya, Animesh Gupta, Andrea Cugno, Luca Deseri, Kaushik Dayal, Dibyendu Das, Shamik Sen, and Mandar M. Inamdar

SUPPORTING INFORMATION

Text S6. DELAUNAY TRIANGULATION AND T1 TRANSITIONS IN OUR MODEL

In our model, the cell connectivity is obtained via Delaunay triangulation from cell centre positions at every time step [1, 2]. The connectivity of the cells is only modified when there is local shear distortion of the tissue, which can be typically relieved by a systematic exchange of neighbour pairs (Figs. S9 and S10). These modification of neighbours that can be interpreted as the so called T1 transitions, the specialised terminology for neighbour exchange in the context of foams and epithelia [3]. T1 transitions that are dependent on shear deformation of cells, have been also observed/modelled in a very recent work by Etournay et al. [4], thus providing biological pointer for distortion driven neighbor changes in a tissue. It may be noted that, in the case of Vertex Models (VM), that have been extensively used for modelling epithelial morphology during development, the T1 transitions typically happen when the edge-length shared by two-cells reduces beyond an arbitrarily chosen critical threshold, upon which the connectivity is updated [3]. The existence of a small edge in a cell, naturally implies the presence of shear strain, and is quite in line with distortion criteria used by Delaunay triangulation (and the corresponding Voronoi tessellation). However, Delaunay triangulation has one advantage of updating the cell connectivity naturally without resorting to potentially ad-hoc assumption used by VM [5, 6].

---

[1] Li B, Sun SX. Coherent motions in confluent cell monolayer sheets. Biophys J. 2014; 107(7):1532–1541.

[2] Pathmanathan P, Cooper J, Fletcher A, Mirams G, Murray P, Osborne J, et al. A computational study of discrete mechanical tissue models. Phys Biol. 2009; 6(3):036001.

[3] Fletcher AG, Osterfield M, Baker RE, Shvartsman SY. Vertex Models of Epithelial Morphogenesis. Biophys J. 2014; 106(11):2291–2304.

[4] Etournay R, Popović M, Merkel M, Nandi A, Blasse C, Aigouy B, et al. Interplay of cell dynamics and epithelial tension during morphogenesis of the Drosophila pupal wing. eLife. 2015; 4:e07090.

[5] Jennings JN. A new computational model for multi-cellular biological systems. University of Cambridge; 2014.

[6] Wyatt TP, Harris AR, Lam M, Cheng Q, Bellis J, Dimitracopoulos A, et al. Emergence of homeostatic epithelial packing and stress dissipation through divisions oriented along the long cell axis. Proc Natl Acad Sci USA. 2015; 112(18):5726–5731.
